# Development of *Aedes aegypti* (Diptera: Culicidae) mosquito larvae in high ammonia sewage in septic tanks causes alterations in ammonia excretion, ammonia transporter expression, and osmoregulation

**DOI:** 10.1038/s41598-019-54413-6

**Published:** 2019-12-13

**Authors:** Andrea C. Durant, Andrew Donini

**Affiliations:** Department of Biology, York University, Toronto, Ontario Canada

**Keywords:** Animal physiology, Entomology, Electrophysiology

## Abstract

Larvae of the disease vector mosquito, *Aedes aegypti* (L.) readily develop in ammonia rich sewage in the British Virgin Islands. To understand how the larvae survive in ammonia levels that are lethal to most animals, an examination of ammonia excretory physiology in larvae collected from septic-water and freshwater was carried out. *A. aegypti* larvae were found to be remarkably plastic in dealing with high external ammonia through the modulation of NH_4_^+^ excretion at the anal papillae, measured using the scanning ion-selective electrode technique (SIET), and NH_4_^+^ secretion in the primary urine by the Malpighian tubules when developing in septicwater. Ammonia transporters, Amt and Rh proteins, are expressed in ionoregulatory and excretory organs, with increases in Rh protein, Na^+^-K^+^-ATPase, and V-type-H^+^-ATPase expression observed in the Malpighian tubules, hindgut, and anal papillae in septic-water larvae. A comparative approach using laboratory *A. aegypti* larvae reared in high ammonia septic-water revealed similar responses to collected *A. aegypti* with regard to altered ammonia secretion and hemolymph ion composition. Results suggest that the observed alterations in excretory physiology of larvae developing in septic-water is a consequence of the high ammonia levels and that *A. aegypti* larvae may rely on ammonia transporting proteins coupled to active transport to survive in septic-water.

## Introduction

The mosquito *Aedes aegypti* (L.) is a medically and economically important species because it is the vector of the arboviruses that cause dengue, Zika, chikungunya and yellow fever^1,2^. The aquatic larvae of *Aedes aegypti* were considered to inhabit clean urban freshwater environments in close proximity to humans; however, over the last few decades, there have been numerous reports of this species exploiting more cryptic, and previously overlooked habitats such as raw sewage and domestic sewage in subterranean septic tanks^3–5^. For example, during dry months in central Nigeria, *A. aegypti* was shown to preferentially breed in containers with septic water over clean water sources^4^. Similar choice assay experiments in a laboratory setting found no differences in the reproductive physiology or selection of oviposition sites by adult *A. aegypti* females provided with freshwater and raw sewage^6^. Habitat expansion of *A. aegypti* to septic tanks was documented in a 1,400 household town of Puerto Rico where it was estimated that septic tanks were yielding tens of thousands of mosquitoes daily^7^. Furthermore, this phenomena is not limited to *A. aegypti* and is not just an issue in areas with regular domestic septic tank usage or poor sanitation practices since the mosquito *Culex quinquefasciatus*, an important vector for West Nile virus, was found in greatest abundance in septic tanks and sewage treatment plants in the Florida Keys^8^. While there appears to be no genetic differentiation between populations of *A. aegypti* emerging from septic/sewage water and freshwater habitats, pupal biomass, adult wing length and nutrient reserves were significantly higher in *A. aegypti* from sewage water compared to man-made and natural freshwater habitats^9–11^. It now appears that septic systems serve as a year-round, permanent refuge for emerging *A. aegypti*, irrespective of rainfall amounts and wet/dry seasons^11^. These findings have serious implications for vector control programs, which traditionally focus efforts on limiting mosquito breeding in freshwater habitats during wet seasons. Furthermore, development of *A. aegypti* larvae in raw sewage can have grave consequences in terms of disease transmission as was shown recently in a study that demonstrated the larvae and pupae can acquire Zika virus in contaminated aquatic systems containing low levels of the virus^12^.

It is clearly beneficial for mosquitoes to seek out protected, predator free breeding habitats rich in organic matter, such as septic tanks, but these habitats also contain ammonia (NH_3_/NH_4_^+^) which is toxic at micromolar concentrations to most animals. Earlier studies examining the tolerance of *A. aegypti* larvae to synthetic sewage containing high levels of ammonium chloride (NH_4_Cl) found that the mean [NH_4_Cl] that is lethal to 50% of the exposed individuals (LC_50_) was between ~2.20–3.57 mmol l^−1^ for different strains of larvae from different geographical regions including Puerto Rico, El Salvador, Africa (3 different strains), India, Sri Lanka, and Malaysia^13,14^. The mean LC_50_ increased to ~12 mmol l^−1^ when larvae were selected over three generations of rearing in high ammonia synthetic sewage. Outside of controlled laboratory studies, high levels of ammonia (~2–4 mmol l^−1^) in septic tanks with actively breeding *Aedes* species (*A. aegypti* and *A. albopictus*) has been documented^5,6^. Therefore, *Aedes aegypti* display a remarkable tolerance to high ammonia and effective surveillance and intervention methods to prevent breeding in septic tanks has been recommended in order to achieve successful mosquito control and disease prevention programs^3,11^.

Ammonia toxicity in animals is caused by a number of mechanisms that have been previously outlined^15–17^. Animals have developed strategies to minimize ammonia toxicity which include mechanisms to sequester and excrete ammonia^15,18,19^. The larvae of *A. aegypti* excrete relatively high levels of ammonium (NH_4_^+^) from the anal papillae (AP)^17,20^. The four AP are sac-like structures that surround the anus and protrude from the terminal segment of the animal. Each AP is comprised of a syncytial epithelium with the apical cell surface directed outwards to the environment, and the basolateral cell surface facing the lumen which is continuous with the haemocoel^21^. This morphology allows for the direct excretion of ammonia into the aquatic environment from the AP, and these organs express a number of ammonia-transporting proteins which have been shown to play a role in ammonia excretion in laboratory reared *A. aegypti*^17,20,22–24^. The AP of laboratory reared *Aedes aegypti* express two vertebrate-like Rhesus (Rh) proteins, AeRh50-1 and AeRh50-2, and two phylogenetically related ammonium transporters (Amts), AeAmt1 and AeAmt2, which group together with functionally similar Amts from plants^17,23,24^. Within the AP epithelium, AeAmt1 and AeAmt2 are localized on the basal and apical sides of the epithelium, respectively, and Rh proteins are localized to both apical and basal membranes^22–24^. Knockdown using RNAi in these studies implicated all four proteins in the process of ammonia excretion and, in part, the regulation of acid-base balance at the AP. Furthermore, Amt and Rh protein mRNA and protein abundances are altered in response to rearing in high environmental ammonia (HEA) conditions, presumably in order to prevent ammonia influx and to continue to facilitate ammonia efflux at the AP against an inwardly directed gradient^24,25^.

Apart from the anal papillae which are only found in some fly (Dipteran) larvae, the main excretory organs of insects are the Malpighian tubules (MT) and rectum (RM) and these have been shown to excrete ammonia in locusts and *Drosophila*^26–28^. In laboratory reared adult *A. aegypti* ammonia is excreted after a blood meal and also after feeding on solutions containing ammonia; however, it is not yet clear which organs are involved^29,30^. In *Manduca sexta*, an Rh-like ammonia transporter (*RhMS*) was shown to have high levels of expression in the Malpighian tubules and gut, and an Rh protein in *Aedes albopictus*, *AalRh50*, was upregulated in the midgut and Malpighian tubules of adult females following blood feeding^31,32^. To the best of our knowledge, no studies have been performed to assess ammonia transport by the Malpighian tubules and rectum of mosquito larvae and despite the importance of the AP in ammonia excretion, one might assume that these organs are also contributing. Furthermore, epithelia of other organs comprising the gastrointestinal tract may aid in regulating ammonia levels during digestion of protein. Specifically, the gastric caeca (GC) which transport ions and other solutes^33–35^, and the anterior and posterior midgut (AMG, PMG) which are important in digestion with the PMG being comprised of cells that resemble the resorbing/secreting cells found in the GC^21^.

Given that sewage contaminated water is being exploited as a suitable habitat by the disease vector mosquito *A. aegypti*, and this species’ remarkable ability to survive in high ammonia environments, the objectives of this study were two-fold; (1) to examine if ammonia transporter expression and rates of ammonia transport in organs of field collected *A. aegypti* larvae from sewage contaminated water and freshwater are different, and (2) to evaluate if differences exist in how laboratory and field collected *A. aegypti* larvae modulate ammonia transporter expression and ammonia transport in organs when exposed to sewage contaminated water. We hypothesized that field collected *A. aegypti* larvae from sewage contaminated water are capable of tolerating high ammonia concentrations by adjusting their physiology in part through altering ammonia transporter (Rh and Amt) expression and function in excretory organs such as the AP, Malpighian tubules and gut.

## Materials and Methods

### Mosquito collection sites

**Figure 1:**
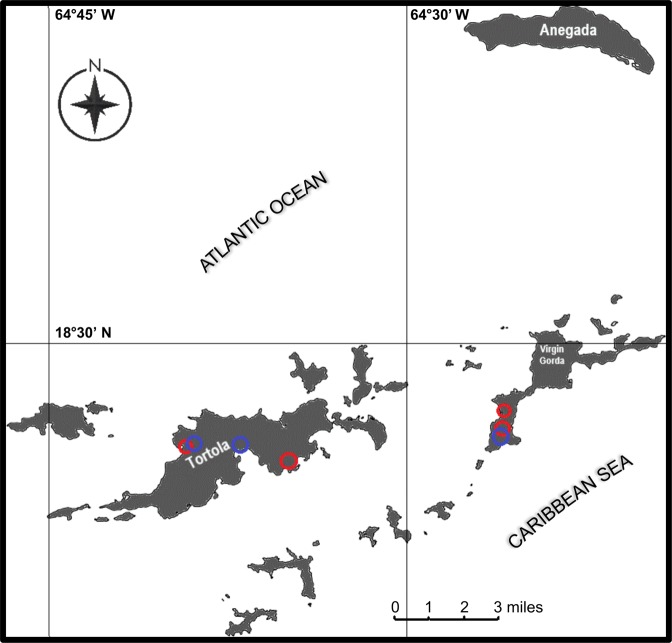
Map of the study area and sites of *A. aegypti* larvae collection within the British Virgin Islands. The location of *Aedes aegypti* larvae collected from septic tanks (6 septic tanks within 4 different areas, red circles) and artificial containers containing freshwater (5 artificial containers within 3 different areas, blue circles) used in the present study are indicated. *A. aegypti* larvae were collected from urbanized sites on the islands of Tortola and Virgin Gorda.

*A. aegypti* larvae were collected from freshwater (FW) artificial containers (4 different artificial containers in three urbanized areas) and septic tanks (6 different septic tanks within 4 areas) in urbanized areas of the British Virgin Islands (B.V.I.) in August and December of 2018 (wet and dry seasons, respectively, Fig. 1). Prior to collection of *Aedes aegypti* larvae from each site, ammonia (NH_3_/NH_4_^+^) test strips (Tetra EasyStrips) were used to confirm high total ammonia levels in septic water compared to freshwater levels. *A. aegypti* was distinguished from other species collected using an Identification Key of Medically Important Mosquito Species developed by the Walter Reed Biosystematics Unit (WRBU, Smithsonian Institution^36^). The septic tanks are domestic sewage systems comprised of a mixture of ‘black water’ which has come into contact with fecal matter from toilets and contains around 90% of a household’s nitrogenous waste as well as the majority of pathogens, and “grey water” generally produced from bath, kitchen and laundry waste^37,38^. The freshwater artificial containers included discarded paint cans, sail cloth (canvas), buckets, and large barrels that had collected rainwater. Samples of raw septic water (5 mL) were collected and immediately frozen at −30 °C for later analysis.

### Mosquito rearing, experimental treatments and organ sample collection

*A. aegypti* larvae were collected from the sites described above using fine nylon mesh nets and were reared to 4^th^ instar in 1 litre of water from their respective collection sites in aerated plastic containers (18 × 14 × 10 cm) outdoors in the shade. Separate containers held under the same conditions were utilized to hatch eggs from a laboratory reared colony in either freshwater or septic water obtained from the collection sites (Laboratory colony from Department of Biology, York University, Toronto, ON, Canada; Details on the establishment of this colony and rearing conditions of the colony have been described in detail in a previous study^24^). All groups of larvae whether field collected or originating from the laboratory colony were fed ¼ teaspoon of Tetrafin goldfish food flakes every other day (Tetra, Melle, Germany). For laboratory sourced larvae two containers of field collected freshwater (from two separate sites where wild larvae were collected) and two containers of septic water (from two separate sites where wild larvae were collected from) were used for hatching ~100 larvae in each container. For wild collected larvae, the larvae were set up in separate containers in their respective collected septic water or freshwater. In the case of wild collected larvae, the number of larvae and rearing density differed depending on how many larvae could be collected from the sites. Upon reaching 4^th^ instar, the field collected and laboratory originating larvae were either fixed in Bouin’s solution and subsequently stored in cold 70% ethanol for immunohistology (see Immunohistochemistry section below), or their organs were dissected on ice in Dulbecco’s phosphate-buffered saline (DPBS, Thermo Fisher Scientific) and stored at −30 °C until processing (see Western blotting section below). Fixed larvae and frozen organ samples were transported to York University, Toronto, ON, Canada on ice with expedited overnight service (FedEx, Mississauga, ON, Canada). Some larvae were also transported live back to Toronto, ON, Canada in their respective rearing water with the same service in order to conduct electrophysiology experiments (see below). These larvae were subsequently held in the laboratory at 26 °C on a 12 h:12 h light:dark cycle, the same parameters utilized to rear the laboratory colony. Importation of *A. aegypti* from the B.V.I. into Canada followed the guidelines of the Government of Canada (Office of the Chief Plant Health Officer, Plant Health and Biosecurity Directorate Canadian Food Inspection Agency).

### Measurement of physicochemical properties of freshwater and septic water from larvae collection sites

The osmolarity of septic water samples (10 µl per sample) from domestic septic tanks was measured using a Precision Systems Osmette II™ osmometer (Thermo Fisher Scientific) which uses a one-step freezing point measurement of osmolality between 0 to 2000 mOsm/L. Total ammonia [NH_3_/NH_4_^+^] in each septic water sample was determined using a colorimetric assay in which a blue indophenol compound is formed^39^. The absorbance spectra were read at 650 nm using a thermo Multiscan Spectrum microplate spectrophotometer (Thermo Electron Co., San Jose, USA) at room temperature. The concentration of free inorganic ions (NH_4_^+^, H^+^, Na^+^, K^+^) were measured using ion-selective microelectrodes (ISMEs, see below). Septic water [Cl^−^] levels were determined using a colorimetric assay which was measured in a spectrophotometer^40^.

### Ion selective micro-electrodes (ISMEs)

ISMEs selective for NH_4_^+^, Na^+^, K^+^ and H^+^ were constructed according to an established protocol^20^. The ISMEs were used to measure hemolymph ion (NH_4_^+^, H^+^, Na^+^, K^+^) activities, Malpighian tubule fluid secretion ion activities, and for Scanning Ion-selective Electrode Technique (SIET) ion flux measurements at the anal papillae. The tips of ISMEs for hemolymph sampling and Malpighian tubule (MT) fluid secretion sampling were coated with polyvinylchloride (PVC, Fluka) in tetrahydrofuran (THF, Fluka) as previously described^20^. The ISMEs were calibrated after every 2 samples in the following solutions (mmol l^−1^): NH_4_^+^, 0.2, 2, 20 NH_4_Cl; Na^+^, 30 NaCl + 270 LiCl and 300 NaCl; H^+^, 100 mmol l^−1^ NaCl and 100 mmol l^−1^ sodium citrate at pH 7.0, 8.0 and 9.0 (pH adjusted by tritration with NaOH or HCl); and K^+^, 0.5, 5, and 50 KCl. The NH_4_^+^ ISMEs for SIET were calibrated in 0.1, 1 and 10 mmol l^−1^ NH_4_Cl.

### Hemolymph and Malpighian tubule fluid secretion sampling

Hemolymph was collected from larvae following a previously established protocol^24^. Hemolymph NH_4_^+^, H^+^, Na^+^, and K^+^ levels of the collected hemolymph droplets under mineral oil were measured as free ion activities using ion-selective microelectrodes (ISMEs). Voltages were recorded with an ML165 pH Amp connected to a PowerLab 4/30 and analyzed in LabChart 6 Pro software (AD Instruments Inc, Colorado Springs, CO, USA).

A modified Ramsay assay described in Misyura *et al*. was used to collect secreted primary urine droplets from MTs of *A. aegypti* larvae^41^. The MTs were left to secrete for 60 mins at room temperature with the distal 1/3 portion bathed in a saline droplet containing 2 mmol l^−1^ of NH_4_Cl. Fluid secretion rates were calculated by dividing the volume of the secreted droplet by the time it took for the droplet to form. ISMEs were used to measure [NH_4_^+^] within the secreted fluid.

### NH_4_^+^ flux measurements using SIET

NH_4_^+^ flux at the anal papillae of *A. aegypti* larvae was measured using the scanning ion-selective electrode technique (SIET) and has been described in detail elsewhere^20,23,24^. Briefly, NH_4_^+^ voltage gradients over an excursion distance of 100 µm were recorded adjacent to the papillae with an ISME selective for NH_4_^+^. Fluxes were measured in the rearing water of each group of larvae (freshwater for FW-reared larvae and septic water for Septic-reared larvae). Flux measurements were taken along the middle to distal portion of the anal papillae at four equally spaced sites. Background voltage gradients were taken 3 cm away from the anal papillae using the same sampling protocol and were subtracted from the voltage gradients recorded at the papillae. For SIET measurements, a single biological replicate (n = 1) is defined as the average flux from 4 repeated measurements at each of the 4 equidistant sites along a single anal papilla from a single larva.

### Body weight and total body moisture

Body weight and body water content were measured from laboratory 4^th^ instar larvae reared in both FW and septic water. The larvae were first placed on tissue paper which allowed all external body surface moisture to be absorbed. The body weight of larvae was recorded (to the nearest microgram, µg) using a UMX2 Automated-S microbalance (Mettler Toledo, Greifensee, Switzerland). Larvae were then placed in a conventional oven at 60 °C for 48 hr to dehydrate and were subsequently reweighed. Total body water content (% of larval weight prior to dehydration) was then calculated using the difference in the mass of the larvae before and after dehydration.

### Western blotting and immunohistochemistry

Quantification of AeAmt1, AeAmt2, and AeRh50 protein abundances in *A. aegypti* larvae using Western blotting has been previously established^24,42^. The present study examined AeAmt1, AeAmt2, and AeRh50 protein abundance in the carcass (CAR), whole gut (WG), and anal papillae (AP) of B.V.I. collected larvae, and in the gastric caecae (GC), anterior midgut (AMG), posterior midgut (PMG), hindgut (HG), Malpighian tubules (MT), and the anal papillae (AP) of laboratory larvae, which were reared in either B.V.I. collected freshwater or septic water. The CAR of larvae in this study is defined as all organs, tissues and cuticle *not* including organs of the alimentary canal and anal papillae, and the WG includes all organs of the alimentary canal (GC, AMG, PMG, HG, MT). Note that GC and AMG are not presented in our normalized protein abundance data for laboratory larvae, as we did not detect ammonia transporter expression in those organs in either FW or septic water reared laboratory larvae. For B.V.I. collected larvae, 3 biological samples were collected from a total of 90 FW and 90 septic water larvae, where 1 biological sample consisted of the CAR, WG or AP of 30 larvae. Protein samples were initially collected and stored in *A. aegypti* saline described previously^43^. Samples of laboratory larvae reared in B.V.I. FW and septic water were similarly collected with 3 and 4 biological samples for FW and septic water, respectively; however, each of these samples were from 50 larvae. Proteins in samples were electrophoretically separated by sodium dodecyl sulphate polyacrylamide gel electrophoresis (SDS-PAGE) as described in detail by Durant and Donini by loading 5 µg (for AP) or 15 µg (for all other organs) of protein (Bradford Assay, Bio-Rad) in RIPA homogenization buffer and 6× loading buffer^25^. Custom-synthesized polyclonal antibodies raised in rabbit against AeAmt1, AeAmt2, and AeRh50s were used at dilutions of 7.46 × 10^−04^, 3.57 × 10^−04^, and 4.29 × 10^−04^ µg µL^−1^ respectively, and have been previously described and used on larvae of *A*. aegypti^22–24^. Due to a high epitope sequence similarity between AeRh50-1 and AeRh50-2, the AeRh50 antisera (designed against AeRh50-1) is presumed to detect both AeRh50-1 and AeRh50-2 protein^22^. Therefore, AeRh50 protein abundance is reported as the combination of both AeRh50-1 and AeRh50-2. Total protein analysis as a loading control was carried out using Coomassie total protein staining^24,44^. Densitometric analysis of AeAmt1, AeAmt2, AeRh50s, and Coomassie total protein was conducted using ImageJ 1.50i software (National Institutes of Health, Bethesda, MD, USA).

AeAmt1, AeAmt2, and AeRh50 immunolocalization in paraffin-embedded cross and transverse sections (5 µm thick) of the *A. aegypti* WG, CAR, and AP was carried out on 10 larvae for each treatment according to an established protocol with the antisera used at dilutions of 9.33 × 10^−03^, 2.86 × 10^−03^, and 6.44 × 10^−03^ µg µL^−1^, respectively^22–24^. Na^+^-K^+^-ATPase (NKA) and the V_1_ subunit of V-type H^+^-ATPase (VA) immunostaining were used as markers for the basolateral and apical membranes, respectively, of organs comprising the alimentary canal^45^. Note that VA is localized to the basolateral membrane in the anterior midgut (AMG) of *A. aegypti* larvae, where NKA immunostaining appears to be absent^45^. A mouse monoclonal anti-α5 antibody for NKA (Douglas Fambrough, Developmental Studies Hybridoma Bank, IA, USA) was used at a 1:10 dilution, and a guinea pig anti-V-type H^+^-ATPase (kind gift from Dr. Weiczorek, University of Osnabruk, Germany) was used at a 1:5000 dilution^23,24,45^. A sheep anti-mouse antibody conjugated to Cy2 and a goat anti-guinea pig antibody conjugated to AlexaFluor 647 (Jackson ImmunoResearch Laboratories, West Grove, PA, USA) at dilutions of 1:500 were used to visualize NKA and VA, respectively. To visualize AeAmt1, AeAmt2 and AeRh50, a goat anti-rabbit antibody conjugated to Alexa Fluor 594 (Jackson ImmunoResearch) at a dilution of 1:500 for all slides was applied^22,23^. Control slides were only incubated with primary immune serum (primary antibody omitted) and processed according to the procedure above. Slides were mounted using with mount media containing DAPI for nuclei staining (ProLong Gold antifade reagent, Life Technologies, Burlington, ON). Fluorescence images were captured on an Olympus IX81 inverted microscope (Olympus Canada, Richmond Hill, ON, Canada) equipped with an X-CITE 120XL Fluorescent Illuminator (X-CITE, Mississauga, ON, Canada)^41,46^. Images were merged using ImageJ 1.50i software (National Institutes of Health, Bethesda, MD, USA).

### Statistical analyses

Data were analyzed using GraphPad Prism 7.00 (GraphPad Software Inc., La Jolla, CA, USA) and were expressed as mean ± S.E.M. All experimental measurements were analyzed using the Student’s *t*-test (*p* < *0.05)* or Two-way ANOVA (Bonferonni’s multiple comparisons test, adjust *p* value < *0.05*) on log transformed values for normalized data and raw values for all other data, as specified.

## Results

### Physicochemical analysis of freshwater and septic water from larvae collection sites

**Figure 2:**
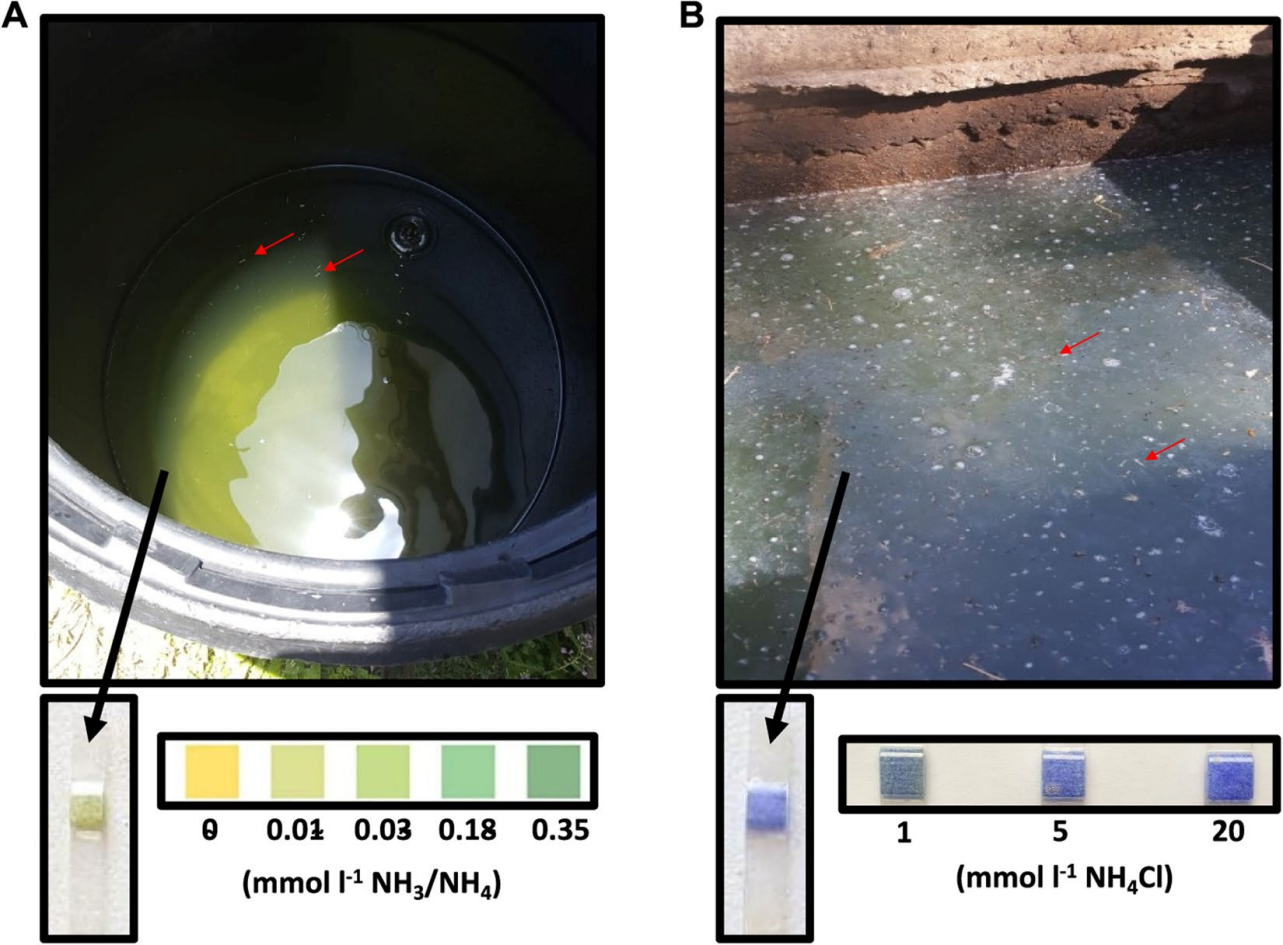
Representative images of a freshwater container and a septic tank containing raw sewage water each with actively breeding *A. aegypti* used in the present study. (**A**) Barrel containing freshwater (FW) and (**B**) septic tank with raw sewage water each containing live *A. aegypti* larvae (indicated by red arrows). Representative ammonia (NH_3_/NH_4_^+^) test strips (Tetra EasyStrips) from one FW site and one septic water site used to estimate ammonia levels are shown below each image (**A**,**B**) along with a control concentration gradient.

*A. aegypti* larvae were collected from artificial containers containing freshwater and residential septic tanks containing sewage water in urbanized regions of the British Virgin Islands (indicated in Fig. 1). Field assessments of total ammonia [NH_3_/NH_4_^+^] in FW containers (Fig. 2A) and septic water from septic tanks (Fig. 2B) using ammonia test strips (Tetra EasyStrips) were carried out. Lower levels of ammonia in FW (~0.015 mmol l^−1^) were detected relative to those in septic water (~5 mmol l^−1^). Ammonia test strips from each of the FW containers and septic tank sites in which *A. aegypti* larvae were collected from in this study are also presented to demonstrate differences in field estimates of total ammonia between FW and septic water (Fig. S1). The osmolarity, concentration of inorganic ions, and total ammonia [NH_3_/NH_4_^+^] levels in septic water collected from three septic tanks was also measured in the laboratory (Table 1). Mean osmolarity (29 mOsm l^−1^) was approximately 10 times less than the hemolymph osmolarity of *A. aegypti* larvae^47^. High levels of total ammonia [NH_3_/NH_4_^+^] and ammonium [NH_4_^+^] were measured in septic water (7.8 and 4.6 mmol l^−1^, respectively), corresponding with crude assessments using ammonia test strips (see above). The mean septic water pH was alkaline (pH 8.5), and moderate amounts of Cl^−^, Na^+^, and to a lesser extent, K^+^, was measured (8.2, 4.3, and 1.2 mmol l^−1^, respectively) (Table 1). Measurements of [NH_4_^+^] in FW from artificial containers containing larvae were much lower (≤0.387 ± 0.015 mmol l^−1^) with osmolarity between 0-5 mOsm l^−1^ (data not shown).

**Table 1:** Concentration of inorganic ions, pH, osmolarity and total ammonia in raw sewage water collected from septic tanks containing developing *A. aegypti* larvae, pupae, and breeding adults.

Osmolarity (mOsm/L)	Total [NH_3_/NH_4_^+^] (mmol/L)	NH_4_^+^ (mmol/L)	pH	Na^+^ (mmol/L)	K^+^ (mmol/L)	Cl^−^ (mmol/L)
29 ± 4.143	7.78 ± 1.217	4.57 ± 0.548	8.48 ± 0.134	4.25 ± 0.438	1.19 ± 0.108	8.24 ± 0.893

Values are shown as mean ± S.E.M. (n = 3 samples of raw sewage). Note: samples of freshwater were not analyzed here, however, freshwater [NH_4_^+^] and osmolarity measurements can be found in text (see Results).

### Hemolymph ion activities, body weight, and body moisture of larvae

**Figure 3:**
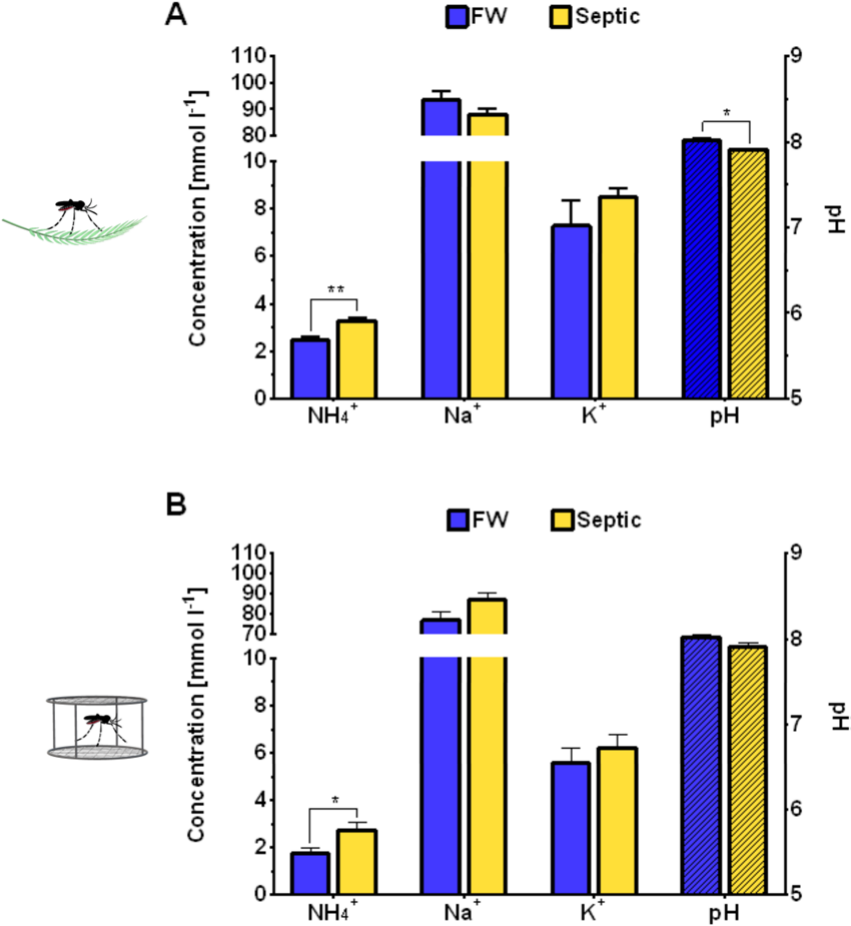
Hemolymph ion and pH levels of wild-collected and laboratory *A. Aegypti* larvae reared in freshwater (FW) and septic water (Septic). Ammonium (NH_4_^+^), sodium (Na^+^), potassium (K^+^) and pH (dashed bars) levels in the hemolymph of (**A**) wild *A. aegypti* larvae (*n* = *6–7* per group) and (**B**) laboratory *A. aegypti* larvae (*n* = *15–17* per group) reared in FW or Septic. Data shown as mean ± S.E.M. Asterisks indicate statistical significance (*p < *0.05*; **p < *0.005*) compared to FW control (*Unpaired, two-tailed t-test*; Adjusted *p* values shown; *Holm-Sidak* correction for multiple comparisons).

**Figure 4:**
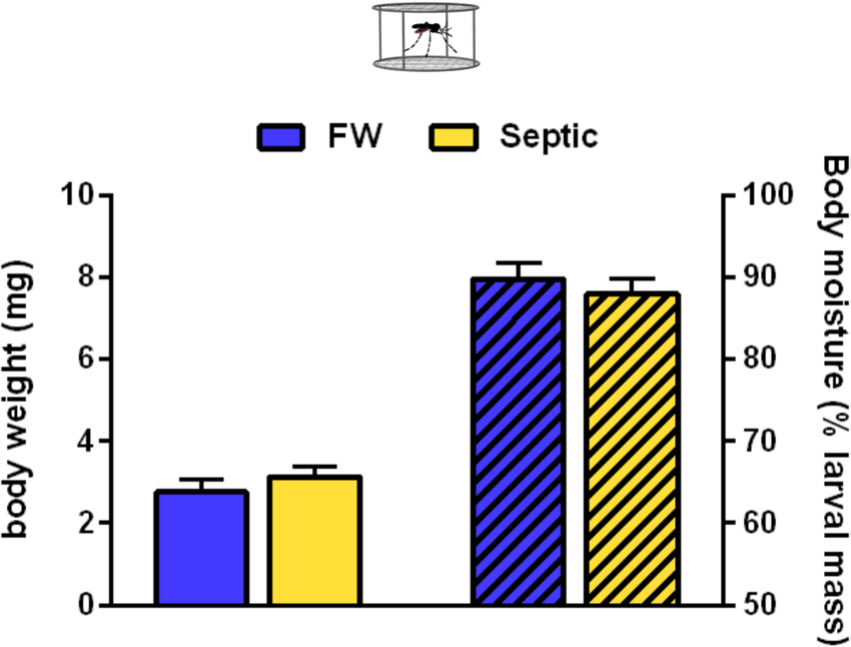
Mean body weight and total body moisture (dashed bars) of laboratory *A. aegypti* larvae reared in freshwater (FW) or septic water (Septic). Data shown as mean ± S.E.M (*n* = *9* for FW, *n* = 9 for Septic). [*Unpaired, two-tailed t-test; p* = *0.35* for body weight, *p* = *0.51* for body moisture].

Hemolymph ion activities and pH of wild collected and laboratory larvae reared in FW and septic water were measured using ISMEs (Fig. 3). A significant increase in hemolymph [NH_4_^+^] was observed in both wild collected and laboratory larvae reared in septic water compared to FW reared larvae (Fig. 3A,B). The pH of the hemolymph of wild collected septic-reared larvae was lower than FW reared larvae (Fig. 3A). There was no difference in hemolymph [Na^+^] and [K^+^] between FW and septic water reared larvae from wild collected or laboratory larvae (Fig. 3). Furthermore, mean body weight (in mg) and total body moisture (between 85–90% of total larval mass) of 4^th^ instar laboratory *A. aegypti* larvae did not differ between FW and septic water treatments (Fig. 4).

### NH_4_^+^ flux at the anal papillae of FW- and septic-reared larvae

**Figure 5:**
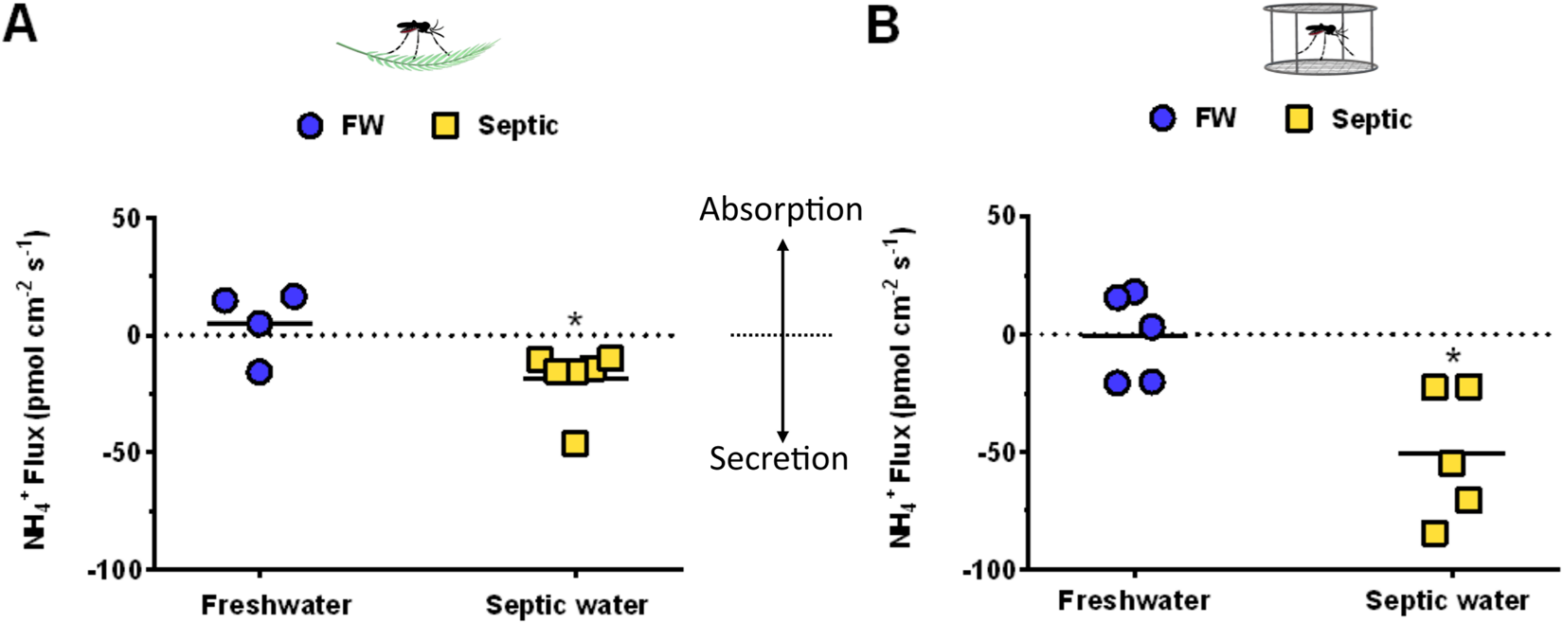
Scanning ion-selective micro-electrode technique (SIET) measurements of NH_4_^+^ flux at the anal papillae of wild-collected and laboratory *A. aegypti* larvae reared in freshwater (FW) and septic water (Septic). (**A**) NH_4_^+^ flux at the anal papillae of wild *A. aegypti* larvae reared in FW or Septic (*n* = 4 for FW, *n* = 6 for Septic), and (**B**) NH_4_^+^ flux at the anal papillae of laboratory *A. aegypti* larvae reared in FW of Septic (*n* = 5 for FW and Septic), measured each in their respective freshwater or septic water baths. NH_4_^+^ flux for each individual animal is shown as a single point, with the mean flux for each group illustrated by a horizontal solid black line. Negative values indicate efflux, or excretion, and positive values indicate influx, or absorption from the external. Data shown as mean ± S.E.M. Asterisks indicate statistical significance (*p < *0.05*) compared to FW control (*Unpaired, two-tailed t-test*).

NH_4_^+^ flux at the AP of FW and septic water reared larvae was measured using SIET (Fig. 5). Flux measurements for FW animals were recorded in a freshwater bath, and flux measurements for septic water collected animals were recorded in a septic water bath. In wild collected *A. aegypti* larvae, a mean absorption (5 ± 7.34 pmol^−2^ s^−1^; n = 3 absorbing and n = 1 secreting) of NH_4_^+^ at the AP of FW larvae, and a mean secretion (−18.5 ± 5.62 pmol^−2^ s^−1^; n = 6 secreting) of NH_4_^+^ at the AP of septic larvae was measured, demonstrating significant differences in the magnitude and direction of NH_4_^+^ transport at the AP between groups (Fig. 5A). Similar observations of significant differences in NH_4_^+^ flux of laboratory *A. aegypti* larvae were seen, with mean NH_4_^+^ flux at the AP of FW larvae being close to zero (−0.9 ± 8.36 pmol^−2^ s^−1^; n = 3 absorbing and n = 2 secreting), and a net NH_4_^+^ secretion (−50.92 ± 12.55 pmol^−2^ s^−1^; n = 5 secreting) at the AP of septic-reared larvae (Fig. 5B). NH_4_^+^ secretion in laboratory septic-reared larvae was approximately 2.75 times greater than NH_4_^+^ secretion in wild septic-reared larvae (*p* = *0.02*9*5;* Two-way ANOVA, Bonferroni’s multiple comparisons test) (Fig. 5).

### NH_4_^+^ activities, NH_4_^+^ transport rates, and fluid secretion rates of MT from FW- and Septic-reared larvae

**Figure 6:**
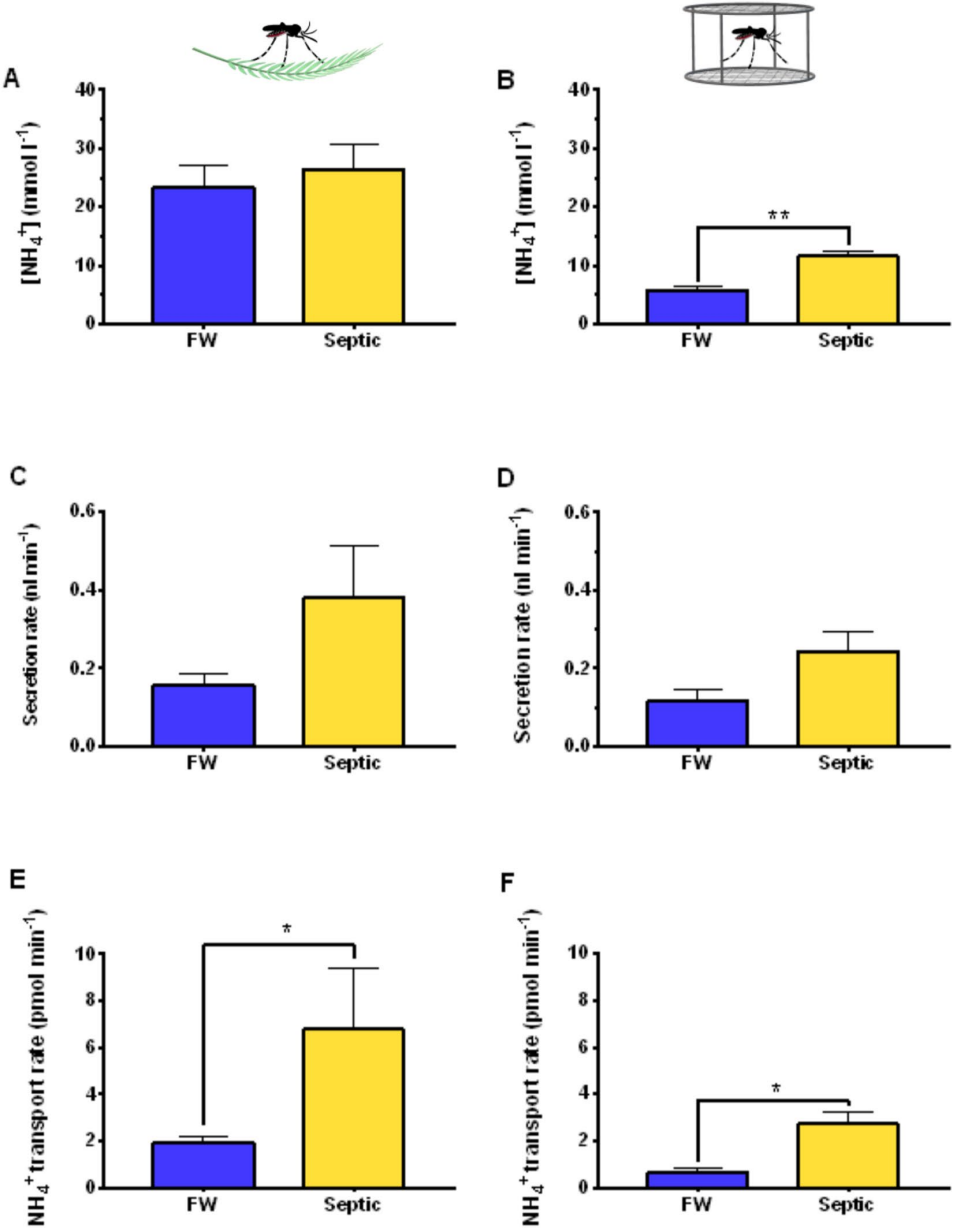
Transepithelial fluid secretion rates, ammonium (NH_4_^+^) concentrations in the secreted fluid, and NH_4_^+^ transport rates of Malpighian tubules from wild-collected and laboratory *A. aegypti* larvae reared in freshwater (FW) or septic water (Septic). NH_4_^+^ concentrations in Malpighian tubule secreted fluid from **(A)** wild *A. aegypti* larvae and **(B)** laboratory *A. aegypti* larvae reared in FW and septic water. Transepithelial fluid secretion rate of the Malpighian tubules from **(C)** wild *A. aegypti* larvae and **(D)** laboratory *A. aegypti* larvae reared in FW and septic water. NH_4_^+^ transport rate by the Malpighian tubules from **(E)** wild *A. aegypti* larvae and **(F)** laboratory *A. aegypti* larvae reared in FW and septic water. Data shown as mean ± S.E.M (*n* = *5–7* wild larvae, *n* = 5 laboratory larvae). Asterisks indicate statistical significance (*p < *0.05*; **p < *0.005*) compared to FW control (*Unpaired, two-tailed t-test*).

The *in vitro* transepithelial fluid ion composition and secretion rates of the MT of wild collected and laboratory *A. aegypti* larvae reared in FW or septic water was examined (Fig. 6). The [NH_4_^+^] in the secreted fluid droplet did not differ between MT from wild collected FW or septic reared larvae (Fig. 6A), however, a significant increase in [NH_4_^+^] in the secreted fluid of MT from laboratory septic reared larvae compared to FW reared laboratory larvae MT was observed (Fig. 6B). Furthermore, the mean [NH_4_^+^] in the secreted fluid of MT from wild collected *A. aegypti* larvae was 23.25 ± 3.9 and 26.4 ± 4.1 mmol l^−1^ for FW and septic water wild larvae, respectively, whilst the mean [NH_4_^+^] in the secreted fluid of MT from laboratory *A. aegypti* larvae was significantly lower, 5.77 ± 0.5 and 11.55 ± 0.9 mmol l^−1^ for FW and septic water laboratory larvae, respectively (*p* = *0.0057* for FW colony vs. FW wild larvae; *p* = *0.0355* for Septic colony vs. Septic wild larvae, Two-way ANOVA, Bonferonni’s multiple comparisons test) (Fig. 6A,B). There was no change in the secretion rates of fluid from the MTs of wild (Fig. 6C) and laboratory (Fig. 6D) larvae between FW and septic treatments, however, significant increases in the transport rates of [NH_4_^+^] within the secreted fluid by MT from laboratory (Fig. 6E) and wild collected (Fig. 6F) larvae was observed with septic water rearing compared to FW groups.

### Ammonia transporter expression and localization in FW- and Septic-reared larvae

**Figure 7:**
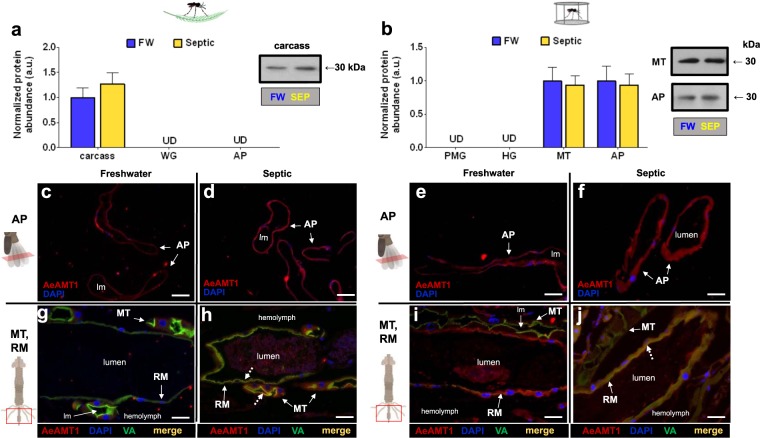
AeAmt1 abundance and immunolocalization in the alimentary canal, anal papillae, and carcass of wild-collected and laboratory *A. aegypti* larvae reared in freshwater (FW) and septic water (Septic). (**a**) AeAmt1 abundance and representative Western blot (right panel) in the carcass of wild *A. aegypti* larvae *(n* = *3)*. (**b**) AeAmt1 abundance and representative Western blot (right panel) in the Malpighian tubules (MT) and anal papillae (AP) of laboratory *A. aegypti* larvae (*n* = 3 FW, *n* = 4 Septic). The abundance of AeAmt1 protein was normalized to total protein (Coomassie protein stain, not shown), and Septic values are expressed relative to the control FW group (assigned a value of 1). Data shown as mean ± S.E.M. [*Unpaired, two-tailed t-test; p* < *0.05]*. Representative transverse sections of anal papillae (AP) showing AeAmt1 (red) immunostaining from (**c**) wild FW-reared larvae, (**d**) wild Septic-reared larvae, (**e**) laboratory FW-reared larvae and (**f**) laboratory Septic-reared larvae. Nuclei are labelled by DAPI (blue) staining. Representative cross sections of the Malpighian tubules (MT) and rectum (RM) showing AeAmt1 (red) immunostaining from (**g**) wild FW-reared larvae, (**h**) wild Septic-reared larvae, (**i**) laboratory FW-reared larvae and (**j**) laboratory Septic-reared larvae. Nuclei are labelled by DAPI (blue) staining. Immunostaining of V_1_ subunit of V-type H^+^-ATPase (VA^)^ is green (**g**–**j**). Co-localization of AeAmt1 with apical V_1_ subunit of V-type H^+^-ATPase is indicated (dashed arrows) in the MT and RM (merge, yellow). Control sections (primary antibodies omitted, not shown) were devoid of red and green staining. Illustrations of the alimentary canal and anal papillae of *A. aegypti* larvae to the left of each immunofluorescence image indicates the region of the cross or transverse section (red rectangles). Lumen, (lm); anal papillae (AP); rectum (RM). Scale bars: 50 µm (**c**–**j**).

Relative protein abundance and immunolocalization of AeAmt1 in FW and septic water reared larvae was examined (Fig. 7). The AeAmt1 monomer (30 kDa) was detected in each organ. In wild collected larvae, AeAmt1 protein expression was only detected in the carcass, and not in the WG and AP of larvae, and no change in AeAmt1 abundance in the carcass was observed between FW and septic water rearing (Fig. 7a). On the other hand, immunohistochemistry did not detect AeAmt1 staining in the carcass but instead revealed AeAmt1 localization in transverse sections of the AP epithelium of FW and septic reared wild larvae (Fig. 7c,d), and in cross sections of the MT and RM epithelia where cytosolic staining in the principal cells and co-localization with VA at the apical membrane of the MT is observed (Fig. 7g,h). In laboratory larvae, AeAmt1 protein was detected in protein homogenates of the MT and AP, whereby rearing in septic water did not affect abundance in comparison to FW controls (Fig. 7b). AeAmt1 was not detected in the PMG and HG through Western blotting. However, immunohistochemistry revealed AeAmt1 localization in transverse sections of the epithelium of the rectum (the distal portion of the HG), where co-localization with VA occurs in septic-reared larvae (Fig. 7i,j), and AeAmt1 localization is also observed in AP cross sections (Fig. 7e,f).

**Figure 8:**
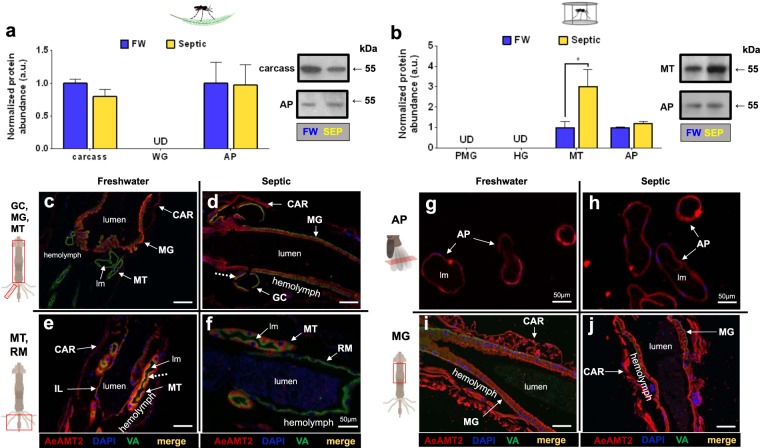
AeAmt2 abundance and immunolocalization in the alimentary canal, anal papillae, and carcass of wild-collected and laboratory *A. aegypti* larvae reared in freshwater (FW) and septic water (Septic). (**a)** AeAmt2 abundance and representative Western blots (right panel) in the carcass and anal papillae (AP) of wild *A. aegypti* larvae *(n* = *3)*. **(b)** AeAmt2 abundance and representative Western blots (right panel) in the Malpighian tubules (MT) and anal papillae (AP) of laboratory *A. aegypti* larvae (*n* = 3 FW, *n* = 4 Septic). The abundance of AeAmt2 protein was normalized to total protein (Coomassie protein stain, not shown), and Septic values are expressed relative to the control FW group (assigned a value of 1). Data shown as mean ± S.E.M. Asterisks indicate statistical significance (*p < *0.05*) compared to FW control (*Unpaired, two-tailed t-test*). Representative cross sections of the carcass (CAR), gastric caecae (GC), anterior and posterior midgut (MG) and Malpighian tubules (MT) and rectum (RM) showing AeAmt2 (red) immunostaining from (**c**–**e**) wild FW-reared larvae, (**d**–**f**) wild Septic-reared larvae. Representative cross sections of the anterior midgut (MG) and transverse sections of anal papillae (AP) showing AeAmt2 (red) immunostaining from (**g**–**i**) laboratory FW-reared larvae and (**h**–**j**) laboratory Septic-reared larvae. Nuclei are labelled by DAPI (blue) staining. Immunostaining of V_1_ subunit of V-type H^+^-ATPase (VA^)^ is green (**c**–**h**). Co-localization of AeAmt2 with V_1_ subunit of V-type H^+^-ATPase is indicated (dashed arrows) (merge, yellow). Control sections (primary antibodies omitted, not shown) were devoid of red and green staining. Illustrations of the alimentary canal and anal papillae of *A. aegypti* larvae to the left of each immunofluorescence image indicates the region of the cross or transverse section (red rectangles). Lumen, (lm); carcass (CAR), gastric caecae (GC), midgut (MG), Malpighian tubule (MT), anal papillae (AP); rectum (RM). Scale bars: 100 µm, unless specified.

AeAmt2 protein (55 kDa monomer) was detected in the carcass and AP of wild collected *A. aegypti* larvae, where AeAmt2 abundance was similar in FW and septic water reared larvae (Fig. 8a). AeAmt2 was not detected in the WG of wild larvae using Western blotting, however, immunohistochemistry revealed AeAmt2 localization in the epithelia of GC, MG, MT, and IL of FW and septic reared larvae (Fig. 8c–f). In all cases, an apical (lumen facing) localization of AeAmt2 was observed in wild collected larvae, with the exception of the MT where both apical and cytosolic staining in the principal cells is shown (Fig. 8e,f). Dashed arrows indicate co-localization of AeAmt2 with apical VA in the MT and GC (Fig. 8d,e). In laboratory larvae, AeAmt2 protein was detected in the MT and AP using Western blotting, whereby AeAmt2 significantly increased in the MT in response to septic water rearing in comparison to FW controls (Fig. 8b). AeAmt2 was immunolocalized within all organs examined, including the epithelium of the AP (Fig. 8g,h), and the midgut (Fig. 8i,j). Localization in the CAR, which includes fat body, muscle, and cuticle (see Methods), was also observed. Similarly, to observations in wild larvae, an apical localization within the epithelia of organs comprising the alimentary canal, and the AP, was observed.

**Figure 9:**
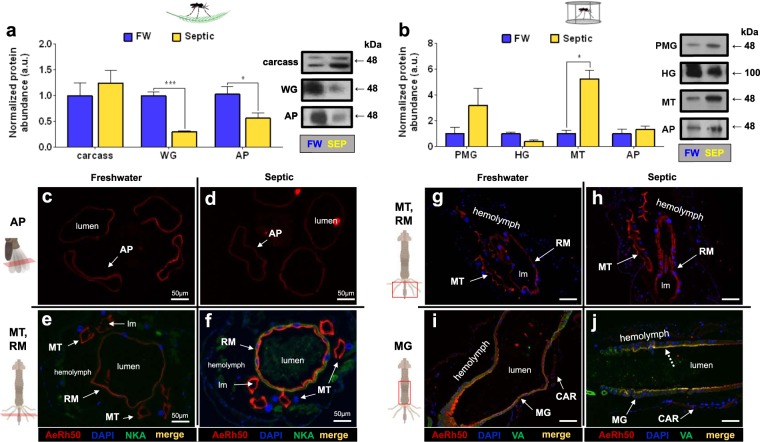
Rh protein (AeRh50) abundance and immunolocalization in the alimentary canal, anal papillae, and carcass of wild-collected and laboratory *A. aegypti* larvae reared in freshwater (FW) and septic water (Septic). (**a)** AeRh50 abundance and representative Western blots (right panel) in the epidermis, whole gut (WG), and anal papillae (AP) of wild *A. aegypti* larvae *(n* = *3)*. **(b)** AeRh50 abundance and representative Western blots (right panel) in the posterior midgut (PMG), hindgut (HG), Malpighian tubules (MT) and anal papillae (AP) of laboratory *A. aegypti* larvae (*n* = 3 FW, *n* = 4 Septic). The abundance of AeRh50 protein was normalized to total protein (Coomassie protein stain, not shown), and Septic values are expressed relative to the control FW group (assigned a value of 1). Data shown as mean ± S.E.M. Asterisks indicate statistical significance (*p < 0.05; ***p < *0.001*) compared to FW control (*Unpaired, two-tailed t-test*). Representative transverse and cross sections of the anal papillae (AP), Malpighian tubules (MT) and rectum (RM) showing AeRh50 (red) immunostaining from (**c**–**e**) wild FW-reared larvae, (**d**–**f**) wild Septic-reared larvae. Representative cross sections of the posterior midgut (MG), Malpighian tubules, rectum (RM), and carcass (CAR) showing AeRh50 (red) immunostaining from (**g**–**i**) laboratory FW-reared larvae and (**h**–**j**) laboratory Septic-reared larvae. Nuclei are labelled by DAPI (blue) staining. Immunostaining of Na^+^-K^+^-ATPase (NKA) (**e**,**f**) and the V_1_ subunit of V-type H^+^-ATPase (VA) (**i**–**j**) are shown in green. Co-localization of AeRh50 with V_1_ subunit of V-type H^+^-ATPase is indicated ^(^dashed arrows) (merge, yellow). Control sections (primary antibodies omitted, not shown) were devoid of red and green staining. Illustrations of the alimentary canal and anal papillae of *A. aegypti* larvae to the left of each immunofluorescence image indicates the region of the cross or transverse section (red rectangles). Lumen (lm), midgut (MG), Malpighian tubule (MT), anal papillae (AP); rectum (RM). Scale bars: 100 µm, unless specified.

AeRh50 protein (48 kDa monomer) was detected in the carcass, WG, and AP of wild collected larvae, whereby AeRh50 protein abundance significantly decreased in the WG and AP of septic water reared larvae compared to FW controls (Fig. 9a). Rh protein immunostaining in transverse sections of the AP epithelium of larvae corresponded with findings from Western blotting, demonstrating a decrease in Rh immunostaining within the epithelium of AP of septic reared larvae in comparison to FW controls (Fig. 9c,d). On the other hand, whilst a significant decrease in Rh protein abundance in response to rearing in septic water using Western blotting was observed, an increase in Rh immunostaining in transverse sections of the MT and RM of septic reared larvae was also observed, compared to FW reared larvae (Fig. 9e,f). Additionally, an increase in NKA immunostaining in the RM of septic water reared larvae compared to FW reared larvae was also detected in these sections. In laboratory larvae, AeRh50 protein was detected in the epithelia of PMG, HG, MT and AP, whereby a significant increase in the MT in response to septic water rearing was observed (Fig. 9b). In all organs the Rh monomer (48 kDa) was detected, with the exception of the HG which displayed only a single band at 100 kDa, the presumed dimer form of the AeRh50 (Fig. 9b, right panel). Immunohistochemistry revealed AeRh50 localization in the MT and RM, where an apparent increase in Rh staining in the MT in septic water reared larvae compared to FW was observed corresponding to the change observed through Western blotting (Fig. 9g,h). Rh staining was also detected in the apical membrane of the MG epithelium (Fig. 9i,j). Similar to AeAmt2 localization, within each organ Rh was localized to the apical membrane (lumen facing) of the epithelium, with the exception of the AP in which localization to a specific membrane could not be determined.

### NKA and VA immunostaining in the AP and RM of FW- and Septic-reared larvae

**Figure 10:**
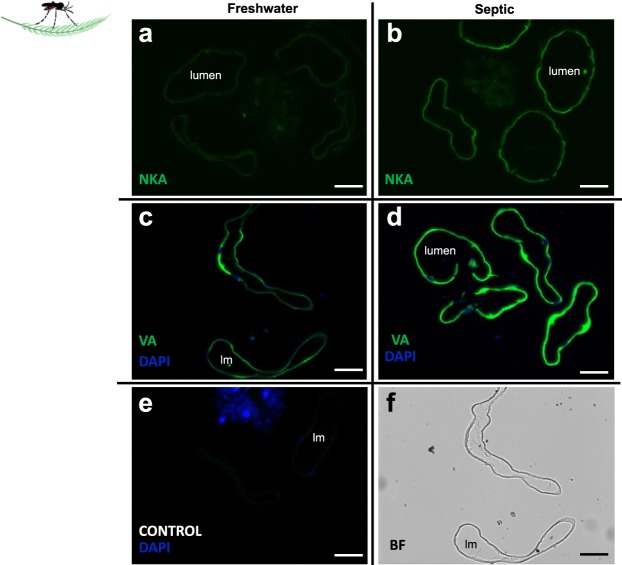
Na^+^-K^+^-ATPase (NKA) and V-type H^+^-ATPase (VA) immunolocalization in the anal papillae (AP) of wild-collected *A. aegypti* larvae reared in freshwater (FW) and septic water (Septic). NKA immunostaining (green) of representative transverse sections of the AP from **(a)** FW-reared larvae and **(b)** Septic-reared larvae. VA immunostaining (green) of representative transverse sections of the AP from **(c)** FW-reared larvae and **(d)** Septic-reared larvae. **(e)** Control sections of AP (CONTROL, primary antibody omitted). DAPI staining of nuclei is in blue. **(f)** Representative bright field (BF) image of AP transverse sections in C. Scale bars: 50 µm. Lumen (lm), anal papillae (AP).

**Figure 11:**
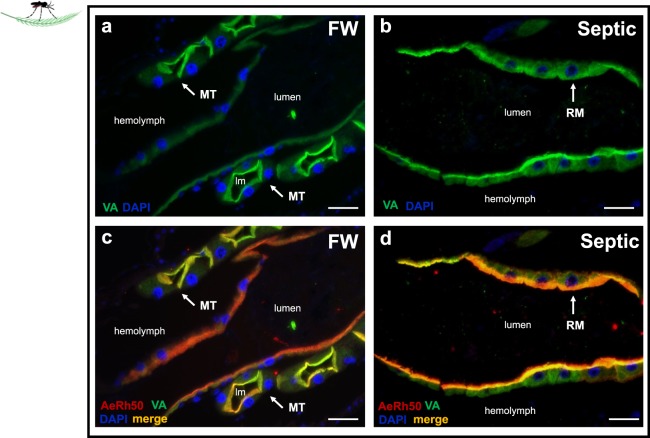
V-type H^+^-ATPase (VA) immunolocalization in the rectum (RM) of wild-collected *A. aegypti* larvae reared in freshwater (FW) and septic water (Septic). VA immunostaining (green) in representative paraffin-embedded cross sections of the **(a)** RM and Malpighian tubules (MT) from FW-reared wild A. aegypti larvae and **(b)** RM of septic-reared wild A. aegypti larvae. Nuclei are labelled with DAPI (blue). **(c)** cross section of MT and RM from (**a**) with apical AeRh50 staining (red) showing colocalization of with VA in MT (merge, yellow) in FW larvae and **(d)** cross section of RM corresponding to (**b**) showing co-localization (merge, yellow) of VA with apical AeRh50 staining (red) in septic larvae. Scale bars: 50 µm. Lumen (lm); rectum (RM), Malpighian tubule (MT).

NKA and VA staining within the AP epithelium of wild collected *A. aegypti* larvae was examined (Fig. 10). Transverse sections of AP from FW and septic water reared larvae showed an increase in immunofluorescence in septic water reared larvae compared to FW groups (Fig. 10a,b). Similarly, compared to FW groups, an increase in VA staining in sections of AP from septic water-reared larvae was observed (Fig. 10e,f). Control slides incubated without primary antibody (NKA or VA antisera) did not show green immunofluorescence, and only nuclei were clearly stained with DAPI in these sections (in blue) (Fig. 10e). A sample bright field image of a transverse section of AP corresponding to Fig. 10c is provided (Fig. 10f). In cross sections of the MT and RM from wild collected larvae, VA staining was localized to the apical membrane in both organs (Fig. 11) in both FW larvae (Fig. 11a,c) and septic-reared larvae (Fig. 11b,d). Co-localization with apical AeRh50 was observed in the MT (Fig. 11c) and RM (Fig. 11d).

## Discussion

### Overview of study and collection sites

Domestic septic tanks and sewage water systems are breeding sites of *A. aegypti*^3–8,10,11^. Physicochemical analyses of septic water report relatively high levels of free ammonia^5,6^ and ammonium is the major toxic component of artificial sewage in the laboratory^13^. With this in mind, the current study examined indicators of ammonia excretion physiology of wild *A. aegypti* larvae collected from septic water and freshwater in an effort to understand how larvae adjust their physiology to inhabit these high ammonia environments. *A. aegypti* from a laboratory colony were also reared in septic water and studied for comparison with the wild collected mosquitoes to validate previous laboratory studies on ammonia excretion physiology when larvae are faced with high external ammonia.

High [NH_4_^+^] and total ammonia levels similar to those reported from numerous other studies were measured from the septic tanks where mosquito larvae were collected in this study^5,6,13^. The pH of the water in these septic tanks of ~8.5 is alkaline compared to that reported in other studies (~7.1 to 7.6) in similar systems in Puerto Rico and Paraná, Brazil^3,6^. This finding may represent an additional challenge to mosquitoes inhabiting these septic tanks in the British Virgin Islands, as ammonia toxicity increases with increasing pH (pKa ~ 9.5) due to increases in the proportion of gaseous NH_3_ which can readily permeate across biological membranes^17,48^. The mean osmolarity of septic water was approximately 10 times lower than that of *A. aegypti* hemolymph, and free [Na^+^], [K^+^], and [Cl^−^] were also lower in the septic water compared to known levels in the hemolymph of larvae^20,41,47^. This indicates that the larvae in these septic tanks are likely to face challenges to ion and water regulation, namely maintaining a hypertonic hemolymph compared to the FW, similar to those of larvae developing in FW but with the additional challenge of high ammonia toxicity.

### Hemolymph composition and [NH_4_^+^] transport by anal papillae

The larvae collected from septic water had a higher hemolymph [NH_4_^+^] and lower pH than those collected from FW. These findings are analogous to results from laboratory studies where larvae reared in 5 mmol l^−1^ NH_4_Cl (High Environmental Ammonia, HEA) had lower hemolymph pH compared to controls reared in FW^25^. Furthermore, larvae from the laboratory colony reared in septic water also had higher hemolymph [NH_4_^+^] compared with those reared in the field collected freshwater. Therefore, the effects of rearing wild and laboratory larvae in septic water and/or HEA on hemolymph pH and [NH_4_^+^] levels are consistent with one another. An elevation of hemolymph ammonia upon rearing in HEA conditions appears to be a common consequence amongst freshwater invertebrates, as well as in the body of some vertebrates^49–52^. Evidently, this increase in hemolymph [NH_4_^+^] and [H^+^] in septic water-reared larvae is not due to dehydration, nor do the larvae in septic water appear to be conserving extra water within intracellular spaces within the body since total body moisture of larvae was similar under septic water and FW rearing conditions. The elevated NH_4_^+^ levels in the hemolymph of mosquito larvae developing in septic water may be leading to acidification as a portion of the NH_4_^+^ dissociates into NH_3_ and H^+^ ^53^.

Previous work demonstrated that laboratory larvae reared in HEA secreted NH_4_^+^ from their anal papillae against an inwardly directed ammonium gradient (e.g. 2.5 mmol l^−1^ NH_4_Cl in bath and ~1.4 mmol l^−1^ NH_4_^+^ in hemolymph)^25^. Here, larvae collected from septic tanks were also secreting NH_4_^+^ against an inwardly directed ammonia gradient from their anal papillae, as were laboratory larvae reared in septic water. Collectively these results indicate that active transport mechanisms are facilitating NH_4_^+^ secretion from anal papillae in both laboratory and wild *A. aegypti* larvae. Since the anal papillae lumen is continuous with the haemocoel of the body^21^, this secretion occurs almost directly from the hemolymph to the external environment and suggests that this is an important physiological strategy to combat the observed elevated hemolymph [NH_4_^+^] levels that these animals experience during development in septic water. Similar findings of increased body ammonia coupled with increased ammonia excretion rates during high external ammonia exposure has been reported in other aquatic freshwater invertebrates including *Caenorhabditis elegans* and *Schmidtea mediterranea*^54,55^.

Laboratory larvae reared in FW (dechlorinated tap-water) secrete NH_4_^+^ from their anal papillae^20,22–24^. Conversely, the majority of wild larvae developing in FW were absorbing NH_4_^+^ at the anal papillae as were the laboratory larvae reared in the FW collected from artificial containers in the British Virgin Islands. The reasons for this difference is not clear but is likely to be driven by either differences in the composition of the water (e.g. dechlorinated municipal tapwater versus field collected water), or the quantity and quality of available nitrogen rich food since the laboratory larvae either secrete NH_4_^+^ or absorb NH_4_^+^ depending on which water they have developed in. Consequently, ammonia absorption by the anal papillae may be a strategy to obtain nitrogen for the synthesis of proteins to support development and growth in a nitrogen deficient environment^32,56^. The results show clear differences between larvae developing in septic water or FW in terms of how their anal papillae are functioning in NH_4_^+^ transport and these differences are likely resulting from differences in the expression and/or activity of transport proteins as discussed below. A number of transport proteins have been studied and implicated in ammonia secretion by anal papillae of *A. aegypti* in the laboratory and a transport model with localization and function of transporters has been presented in numerous previous studies^22–25^.

The anal papillae of septic water wild collected larvae had lower Rh protein abundance and Rh-like immunostaining intensity compared to FW collected larvae. These results were consistent with previous findings from laboratory larvae reared in HEA^24^. Since Rh proteins are thought to conduct gaseous NH_3_ bidirectionally and are dependent on the *P*_NH3_ gradient across the biological membrane in which they are expressed, it was proposed that the decrease in Rh protein expression in response to HEA is a mechanism to limit NH_3_ influx caused by an inwardly directed gradient in a high ammonia environment^57–59^. The immunostaining intensity of the primary active pumps, NKA and VA in the anal papillae epithelium of larvae collected from septic water was higher than larvae collected from FW, largely suggesting that the expression of these pumps are higher in the anal papillae of wild septic water collected larvae. These findings parallel that of a previous study on laboratory *A. aegypti* larvae reared in HEA, where VA activity was approximately 3 times higher in the anal papillae of these HEA larvae compared to control larvae reared in FW^25^. These results are also consistent with an actively driven secretion of NH_4_^+^ from the anal papillae in high ammonia water.

The expression of the putative ammonia transporters AeAmt1 and AeAmt2 has also previously been demonstrated in the anal papillae of laboratory *A. aegypti*. Here, while AeAmt1 could not be detected in protein homogenates of the AP of wild collected mosquitoes through Western blotting, AeAmt1-like immunostaining was evident and consistent with that reported for laboratory mosquitoes^23^. On the other hand, AeAmt2 expression in the AP of wild mosquitoes was detected with both western blotting and immunohistochemistry, and consistent with that reported for laboratory mosquitoes^24^. The expression and abundance of AeAmt1 and AeAmt2 in the AP of wild larvae was unaffected by development in septic water versus development in FW which suggests they have an important role in facilitating NH_4_^+^ secretion from the AP regardless of external ammonia levels. AeAmt1 protein abundance increases 48 hours after transferring laboratory larvae to HEA and then returns to levels that are equal to controls after 7 days in HEA^24^. Therefore, in both wild and laboratory larvae AeAmt1 appears to be particularly important for NH_4_^+^ secretion by anal papillae. On the other hand, AeAmt2 protein abundance in the AP was shown to decrease in laboratory larvae transferred to HEA when this was assessed at 48 hours and 7 days after transfer to HEA^24^. Hence, in wild larvae, AeAmt2 appears to play a greater role in ammonia secretion by anal papillae than it does in the laboratory larvae when external levels of ammonia are high. Overall, our results with the wild larvae collected from septic water and FW in the British Virgin Islands largely support the ammonia transport models proposed by our previous work on laboratory reared *A. aegypti*. Furthermore, given the similarities of the results from septic water and laboratory HEA rearing we can conclude that ammonia is the major component of septic water and is the principle factor leading to our observations on alterations in ammonia excretory physiology of *A. aegypti* larvae.

### Putative ammonia transporter expression in the gut and malpighian tubules

Ammonia transporter expression and localization within the osmoregulatory and excretory organs of mosquitoes has presently only been examined in the blood-feeding adult female mosquito *Aedes albopictus* (*AalRh50*) whereby *AalRh50* is elevated in the gut following a blood meal^31^. In the present study we have mapped the expression and localization of AeRh50s, AeAmt1 and AeAmt2 in the gut and Malpighian tubules of wild and laboratory mosquitoes that developed in field collected FW and septic water.

Our previous studies on protein homogenates of anal papillae of laboratory *A. aegypti* larvae detected bands of ~30, ~55 and ~48 kDa for AeAmt1, AeAmt2 and AeRh50s, respectively^22–24^. Here, AeRh50 protein was detected as a ~48 kDa band in whole gut (including MTs) homogenates from wild larvae with lower abundance in the larvae collected from septic water compared with those from FW. Immunostaining of gut sections revealed qualitatively greater intensity of staining in the Malpighian tubules and rectum compared with other regions of the gut. Furthermore, the intensity of AeRh50 immunostaining in the MTs and rectum appeared to be greater in the wild larvae that developed in septic water. It is possible that changes in AeRh50 abundance in other organs in the WG extracts (other than the MTs and rectum) are responsible for the observed decrease of protein abundance through Western blotting, particularly the midgut which is comparatively large. In homogenates of posterior midgut and Malpighian tubules of laboratory larvae that developed in field collected FW or septic water, AeRh50 protein was detected as a ~48 kDa band, whereas in the hindgut a band of ~100 kDa was detected which may be representative of dimers of AeRh50s. The Rh50 proteins are thought to function as homotrimers which would yield a band of ~150 kDa, thus the significance of the ~100 kDa band in the hindgut protein homogenates is unclear at this time^60^. Greater abundance of AeRh50 was detected in the MTs of larvae that developed in septic water relative to FW, but there was no difference in AeRh50 protein abundance in the hindgut (including rectum) between the two conditions. Furthermore, an apical localization of AeRh50 protein was found in the MT, RM and midgut epithelia.

In the MTs, AeRh50 is co-localized with apical VA which parallels the localization of AeRh50 with VA in the AP of *A. aegypti* larvae^22^. This suggests the possibility of an ammonia trapping mechanism in the MT for ammonia secretion in primary urine, as has been described in the AP^22^. An ammonia trapping mechanism has been proposed for ammonia secretion in the MT of *M. sexta* where an Rh protein would work in conjunction with VA^32^. AeAmt1 was detected as a ~30 kDa band in MT protein homogenates where it was localized to the cytosol of epithelial cells and expression was not affected by the water in which the larvae developed. AeAmt2 was detected as a ~55 kDa band in MT protein homogenates and expression was higher in MTs of laboratory larvae that developed in septic water. AeAmt2 was localized to the cytosol of MT epithelial cells. Since both AeAmts are expressed in MT epithelial cells they are likely to play a role in ammonia transport by the MTs but their apparent cytosolic localization makes it difficult to speculate how.

Consistent with the involvement of these transporters in ammonia transport, NH_4_^+^ was present in secreted fluid of MTs at concentrations ranging from ~5 to ~25 mmol l^−1^ depending on treatment when they were bathed in saline containing 2 mmol l^−1^ NH_4_^+^. These levels are much higher than those reported for *Drosophila* MTs which required bathing in 20 mmol l^−1^ NH_4_^+^ to reach similar concentrations in the secreted fluid^28^. This indicates that MTs of *A. aegypti* larve are better equipped to clear ammonia from the hemolymph and can do so against a gradient. Although fluid secretion rates of MTs from wild and laboratory mosquitoes were similar, the secreted fluid of MTs from wild larvae contained greater concentrations of NH_4_^+^ than those from laboratory larvae which was indicative of the higher rates of NH_4_^+^ transport in MTs of wild larvae. Furthermore, the MTs of larvae that developed in septic water had even higher NH_4_^+^ transport rates regardless of whether they were from wild or laboratory larvae. This increased NH_4_^+^ transport rate appeared to be driven by increased rates of fluid secretion that were almost double but not statistically different from those of MTs of larvae that developed in FW. It is likely that the observed increased expression of AeRh50s and AeAmt2 in the MTs of larvae that develop in septic water aid in transporting significantly more NH_4_^+^ over time to clear more NH_4_^+^ that accumulates in the hemolymph of these larvae.

The rectum (RM) of *A. aegypti* larvae is important for the reabsorption of Na^+^, K^+^ and Cl^−^ prior to the excretion of a dilute urine, and basolateral NKA is a key ion-motive pump in energizing ion reabsorption in aquatic dipterans^45,61^. Although pinpointing the expression of VA in the RM of *A. aegypti* has proven challenging prior to this study, here we report that VA is on the apical side of the RM epithelium of FW and septic reared wild and laboratory *A. aegypti* larvae (Fig. 11)^45,62^. Relatively high levels of ammonia (~60 mmol l^−1^) were measured in the rectal lumen of *M. sexta*, along with high mRNA expression of an Rh protein, RhMS in tissue homogenates of the rectum^32^. It was postulated that the function of NKA and RhMS is to confine ammonia within the rectal lumen for excretion; however, the localization of RhMS protein in the rectal epithelium remains to be determined. In the RM of *A. aegypti* larvae AeRh50 proteins were localized to the apical side of the rectal epithelium and both AeRh50 and VA immunostaining intensity qualitatively increased in wild larvae developing in septic water. The arrangement of these transporters is the same as that presented here for the MTs and for the AP with AeRh50 colocalized with VA on the apical side of the epithelium. Therefore, the RM could sequester ammonia from the hemolymph through ammonia trapping while preventing the ammonia secreted into the primary urine by the MTs from being reabsorbed back into the hemolymph prior to excretion.

## Conclusion

*Aedes aegypti* larvae were readily observed developing in septic tanks in the British Virgin Islands during both rainy and dry seasons. The ammonia levels in water collected from these septic tanks was ~8 mmol l^−1^, levels that would kill most aquatic animals^19,63,64^. In order to understand the underlying physiology that allows *A. aegypti* to inhabit this high ammonia refuge, an examination of systemic physiological parameters, the ammonia excreting capacity, and the expression of ammonia transporters of larvae from septic tanks and freshwater was conducted. A comparison with laboratory sourced *A. aegypti* and previous laboratory studies conducted with high environmental ammonia (HEA) show that physiological differences between larvae developing in septic water versus freshwater are largely driven by the ammonia in septic water. Our results reveal a physiological triad of organs including the Malpighian tubules, rectum and anal papillae equipped to deal with the transport of ammonia for excretion. These organs are armed with ammonia transporting proteins and their expression can be adjusted by external ammonia levels leading to measurable differences in ammonia transport function which favours excretion. We therefore conclude that *Aedes aegypti* larvae possess inherent, inducible mechanisms for ammonia excretion in high ammonia environments which, at least in part, permits them to inhabit and complete development in septic water.

## Supplementary information

Supplementary information

## Data Availability

The datasets generated and analyzed during the current study are available from the corresponding author upon reasonable request.
