# Supplementary material for: Development of Aedes aegypti (Diptera: Culicidae) mosquito larvae in high ammonia sewage in septic tanks causes alterations in ammonia excretion, ammonia transporter expression, and osmoregulation
Source: Sci Rep. 2019 Dec 13;9:19028. doi: 10.1038/s41598-019-54413-6 (PMC6911005; doi:10.1038/s41598-019-54413-6)
Supplement: Supplementary file 1 — Supplementary information [file 41598_2019_54413_MOESM1_ESM.docx]

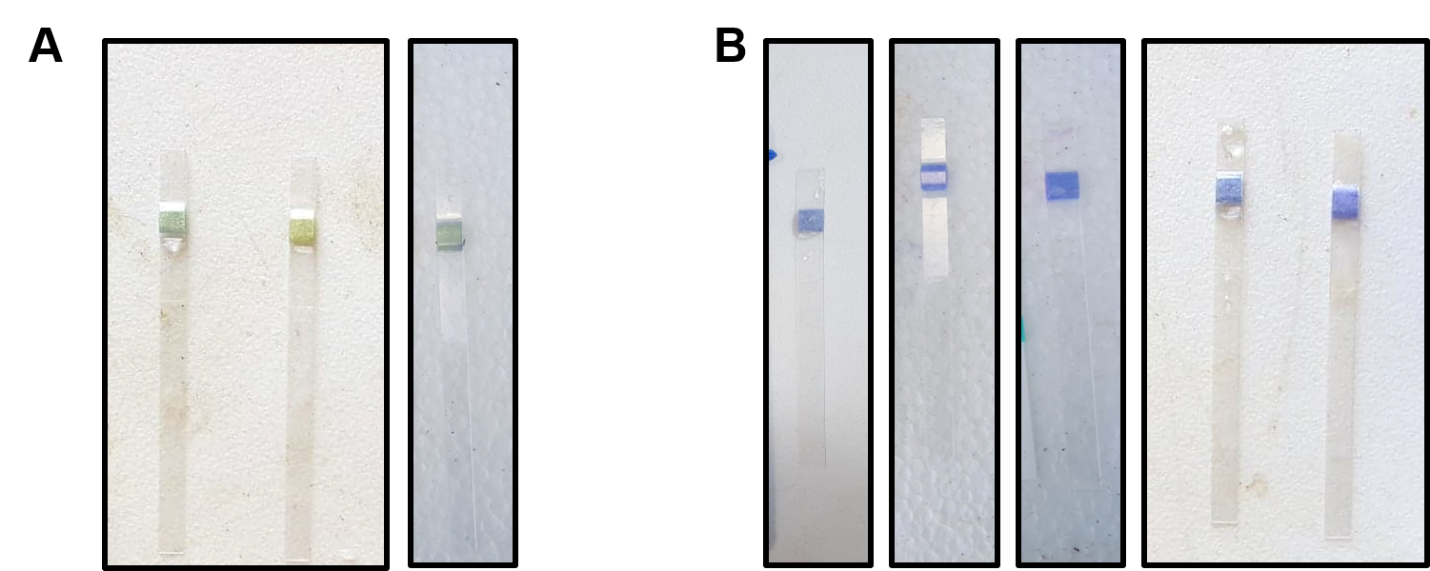


**Supplementary Figure S1.** Ammonia (NH_3_/NH_4_^+^) test strips (Tetra EasyStrips™) used to estimate total ammonia levels in **(A)** freshwater sites and **(B)** septic water sites in which wild *A. aegypti* larvae were collected from and used in this study.
